# Nanoencapsulation Strategies for Active Compounds Delivery

**DOI:** 10.3390/nano12081319

**Published:** 2022-04-12

**Authors:** Claudia Carbone, Carla Caddeo, Teresa Musumeci

**Affiliations:** 1 Laboratory of Drug Delivery Technology, Department of Drug and Health Sciences, University of Catania, Viale A. Doria 6, 95125 Catania, Italy; 2 Department of Life & Environmental Sciences, University of Cagliari, Via Ospedale 72, 09124 Cagliari, Italy

Nanoencapsulation strategies, including the possibility to deliver natural compounds, synthetic molecules, or other actives (viruses) for the treatment of different human diseases, represent a hot topic of great interest. This Special Issue gathers results and new insights on different nanoencapsulation strategies, which specifically focus on lipid nanoparticles [1,2,3,4], polymeric nanoparticles [5,6], vesicular systems [7,8,9], and nanohydrogel [10].

The delivery of powerful natural antioxidants by solid lipid nanoparticles (SLNs) and nanostructured lipid carriers (NLCs) has been described, demonstrating the ability of lipid nanoparticles in improving the drug bioavailability of Diosgenin [1], Astaxanthin [2], N-palmitoylethanolamide [3], and Ferulic Acid, in combination with Lavender essential oil [4], thus offering promising strategies for the cutaneous, ophthalmic, and systemic treatment of diseases involving inflammation.

Some of these, such as ferulic acid, have also been investigated in vesicular systems, demonstrating the possibility to exploit multiple emulsions as an innovative and efficient vehicle for its cutaneous application [5]. Nanoemulsions represent versatile delivery systems, as demonstrated by the study regarding the ophthalmic delivery of the traditional corticosteroid Triamcinolone acetonide [6]. The preparation method of the nanoemulsion was exploited to prepared nanohydrogel for the delivery of Oncolytic viruses as potential anti-cancer treatment [7].

Among vesicular systems, liposomes have also been proposed for the delivery of the natural anti-inflammatory hydroxycitrate [8].

Polymeric nanoparticles with a special focus on nanocapsules have been reviewed [9]. In this field, an interesting approach was developed investigating the delivery of Curcumin by both lipid and polymeric nanoparticles to exploit its anti-inflammatory and antioxidant activity via intranasal administration [10].

Readers will certainly find other interesting aspects exploiting the possibility to study the implication of the different strategies presented in this Special Issue on the delivery of active compounds.

We hope that all readers will enjoy it.
